# The impact of mass screening and treatment interventions on malaria incidence and prevalence: a retrospective analysis of a malaria elimination programme in eastern Myanmar, and systematic review and meta-analysis

**DOI:** 10.1186/s12936-025-05392-9

**Published:** 2025-05-08

**Authors:** Jade D. Rae, Angela Devine, Chanapat Patekkham, Aung Myint Thu, Gilles Delmas, Daniel M. Parker, Richard J. Maude, Jacher Wiladphaingern, Ladda Kajeechiwa, May Myo Thwin, Saw Win Tun, Julie A. Simpson, François H. Nosten

**Affiliations:** 1 Bernhard Nocht Institute for Tropical Medicine (BNITM), Research Group Neglected Diseases and Envenoming, Hamburg, Germany; 2 Shoklo Malaria Research Unit (SMRU), Mahidol-Oxford Tropical Medicine Research Unit (MORU), Faculty of Tropical Medicine, Mahidol University, Mae Sot, Thailand; 3 Mahidol-Oxford Tropical Medicine Research Unit (MORU), Faculty of Tropical Medicine, Mahidol University, Bangkok, Thailand; 4 Centre for Epidemiology and Biostatistics, Melbourne School of Population and Global Health, The University of Melbourne, Melbourne, Australia; 5 Global and Tropical Health Division, Menzies School of Health Research, Charles Darwin University, Darwin, Australia; 6 Population Health and Disease Prevention, University of California-Irvine, Irvine, CA USA; 7 Epidemiology and Biostatistics, University of California-Irvine, Irvine, CA USA; 8 Centre for Tropical Medicine and Global Health, Nuffield Department of Medicine, University of Oxford, Oxford, UK; 9 The Open University, Milton Keynes, UK

## Abstract

**Background:**

Targeted interventions are often needed to accelerate malaria elimination efforts. Mass screening and treatment (MSAT) involves testing all eligible and consenting individuals in an area for malaria and treating all positive individuals simultaneously. However, there are concerns regarding the impact of MSAT. This study evaluates the impact of MSAT on malaria incidence in Karen State, Myanmar, using routine surveillance data, and investigates the impact of MSAT in other settings through a systematic review and meta-analysis.

**Methods:**

To investigate the impact of MSAT in Karen State, we retrospectively analysed routine malaria surveillance data collected in 10 villages where MSAT was done in 2018. Pre- and post-MSAT malaria incidences were compared, and a negative binomial mixed-effects model was used to estimate the relative change in monthly incidence for each additional year since MSAT.

To investigate the impact of MSAT in other settings, we searched Scopus, Ovid MEDLINE, and Web of Science (end date 11th July 2022) for studies assessing the impact of MSAT interventions on the incidence or prevalence of malaria infections. Studies were summarized, and a random-effects meta-analysis was performed on studies grouped according to study design and the comparator used to assess the impact of MSAT.

**Results:**

In the 10 villages in Karen State, there was an overall reduction in *P. falciparum* incidence following MSAT (Incidence Rate Ratio 0.37; 95% CI: 0.19, 0.73). However, this is likely due to the ongoing impact of early diagnosis and treatment services offered in these communities, as shown by an overall reduction in incidence in the surrounding area. Results from nine studies identified in the systematic review demonstrate the variable impact of MSAT, which is likely influenced by a variety of factors, including intervention coverage and uptake, baseline malaria endemicity, and methods used for MSAT delivery.

**Conclusions:**

This retrospective analysis and systemic review highlights the complexities behind the success of targeted interventions for malaria elimination. While these interventions are important drivers for achieving elimination goals, particularly in high-burden settings, it is important that various factors be considered when determining their suitability and how to optimize implementation.

**Supplementary Information:**

The online version contains supplementary material available at 10.1186/s12936-025-05392-9.

## Background

Early diagnosis and treatment of malaria cases is a cornerstone of elimination efforts. In some areas, continued early diagnosis and treatment uptake is sufficient to achieve local elimination. Still, in many regions, targeted interventions, including mass drug administration (MDA) and mass screening and treatment (MSAT), are required to overcome elimination barriers. These targeted malaria interventions aim to eliminate malaria cases in areas defined as “hotspots” based on high incidence or prevalence estimates or in areas where malaria transmission is low but residual transmission has prevented elimination [1].

During MDA, the entire eligible, consenting population is treated without diagnosis. This approach has proven effective in rapidly reducing *Plasmodium falciparum* incidence and prevalence in several studies across Southeast Asia and sub-Saharan Africa [1–4]. However, concerns that MDA could put selective pressure on parasites to develop artemisinin resistance have limited endorsement for its use despite evidence to suggest it does not [5–8]. As an alternative, during MSAT (also referred to as foci screening and treatment (FSAT)), the entire eligible, consenting population is screened, typically using rapid diagnostic tests (RDTs) or ultra-sensitive RDTs (uRDTs), and all positive cases are treated, reducing the number of treatments administered. However, the low sensitivity of these diagnostic tools means low parasite-density infections remain undetected [9, 10]. This limits the impact of MSAT on ongoing transmission, particularly in areas of low transmission where there is a higher proportion of subpatent infections which remain undetected and untreated [11].

In 2014, the Malaria Elimination Task Force (METF) programme deployed a network of malaria posts in villages across Karen State, Myanmar, which continue to provide access to early diagnosis and treatment [12]. In the period between 2014 and 2018, MDA was deployed by the METF programme in villages with a high prevalence of *P. falciparum* malaria detected in qPCR surveys (40% *Plasmodium* spp. prevalence overall and *P. falciparum* detected in 20% of *Plasmodium*-positive individuals). Despite the confirmed effectiveness of MDA interventions [2, 13], in 2018, the Myanmar National Malaria Control Programme (NMCP) ceased approval of MDA for *P. falciparum* malaria. As a result, from 2018 onwards, the METF programme replaced MDA with MSAT.

To date, three systematic reviews have been published on the effectiveness of MSAT in reducing malaria transmission [14–16]. However, community-wide MSAT delivery was not included in the selection criteria in these reviews, while the objective of this review is to assess the effectiveness of MSAT interventions delivered at the village level within a defined population. This study has two primary aims: firstly, to summarize the use and impact of MSAT interventions conducted by the METF programme on village-level *P. falciparum* incidence in Karen State, Myanmar, and secondly, to investigate and compare the impact of MSAT in other settings through a systematic review and meta-analysis of relevant literature.

## Methods

### Study design

To evaluate the association between MSAT and *P. falciparum* incidence, this retrospective study used surveillance data collected from 10 METF malaria posts where MSAT was deployed. The results from this analysis were then combined in a meta-analysis with studies identified from a systematic review of village-level MSAT interventions. Accordingly, the methods and results of this study are separated into two parts: (1) the analysis of MSAT in the METF programme and (2) the systematic review and meta-analysis of published MSAT studies.

### MSAT in the METF programme

**Fig. 1:**
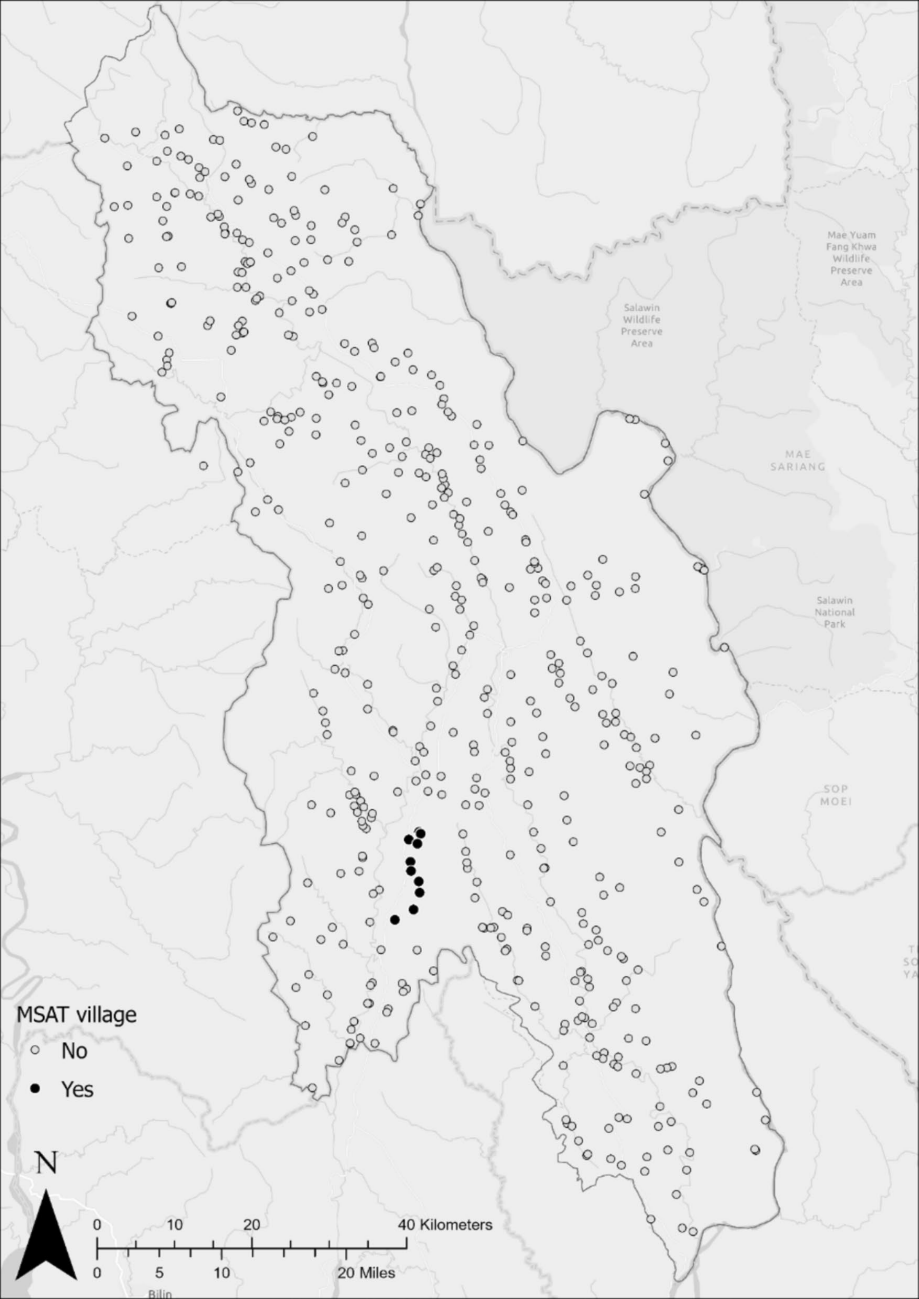
Malaria posts that received an MSAT intervention. Malaria posts are coloured according to whether they did (*black*) or did not (*grey*) receive an MSAT intervention delivered by the METF programme

In 2018, the METF program conducted one round of MSAT in 10 villages (Fig. 1) in response to persistently high *P. falciparum* incidence (determined on a case-by-case basis) detected in weekly data collected by the malaria posts. MDA was conducted in two of the 10 villages approximately three years prior to MSAT.

During MSAT, all consenting village residents were tested using uRDTs (NxTek™ Eliminate Malaria Pf, manufactured by Abbott Diagnostics Korea Inc.) and standard *Plasmodium falciparum*—*Plasmodium vivax* RDTs (BiolineMalaria Ag P.f/P.v, Abbott Diagnostics Korea Inc). All consenting residents of the villages were eligible for inclusion except children younger than 6 months, individuals with an allergy to anti-malarial drugs, individuals who had received treatment for *P. falciparum* in the previous 7 days, and women in their first trimester of pregnancy. Individuals positive for *P. falciparum* by uRDT were treated with one round of dihydroartemisinin-piperaquine (7 mg/kg dihydroartemisinin, 55 mg/kg piperaquine) administered once per day for three consecutive days plus a single dose of primaquine (0.25 mg/kg). Pregnant women in their second or third trimester of pregnancy and breastfeeding women were eligible to receive dihydroartemisinin-piperaquine but not primaquine. Individuals positive for *P. vivax* by uRDT were treated with chloroquine once daily for three consecutive days (10 mg/kg on days 1 and 2, 5 mg/kg on day 3). The dormant liver stage of *P. vivax* parasites should typically be eliminated by treatment with an 8-aminoquinoline drug (radical cure), but this can result in haemolysis in glucose-6-phosphate-dehygrogenase (G6PD) deficient individuals [17–19]. Due to the presence of G6PD deficiency in the Karen population [17] and the absence of reliable G6PD testing during MSAT interventions, radical cure was not administered in this study.

Prior to MSAT, community engagement meetings were organized with the village leader and villagers to discuss the purpose of MSAT and how screening and treatment would be delivered. Larger settlements, typically consisting of military camps, logging camps, or mining sites near the target village, were also approached for inclusion in the MSAT intervention.

#### Statistical methods

For each month from when MSAT was conducted, *P. falciparum* and *P. vivax* incidence were calculated from weekly surveillance data as the number of cases over person-time exposed at the malaria post for the entire period of malaria post functioning. Person-time exposed was calculated at the malaria post level using village census data collected at the time of MSAT.

To estimate the relative change in *P. falciparum* and *P. vivax* monthly incidence for each additional year since MSAT, negative binomial mixed-effects modelling was performed with the population at each malaria post per month as the person-time denominator. Separate models were fit for *P. falciparum* and *P. vivax* to allow for differing impacts of MSAT on the incidence of the two malaria species. A random intercept was included for malaria posts to account for baseline village-level differences in incidence not accounted for in the models, a random slope was included for the number of years since MSAT, as well as its quadratic term to account for both linear and non-linear changes in temporal patterns between malaria posts. Fourier terms were included in the regression model to account for seasonality in malaria transmission over time. Some important confounders, including malaria post functionality, could not be accounted for in the model. Measures of malaria post functionality were collected during monitoring and evaluation assessments conducted at different points in time. Therefore, these indicators of functionality may not reflect the functionality of malaria posts over time. Additionally, the malaria posts opened at different time points, so they contributed variable amounts of data to the before- and after-MSAT periods. This could not be accounted for easily due to the dynamics between the date of malaria post opening, the date of MSAT delivery, and the date of malaria post closure. Statistical analyses were performed using R (version 3.6), and mapping was performed in ArcGIS Pro (version 2.5).

### Systematic review

#### Search strategy and selection criteria

To be eligible for inclusion, studies needed to describe the implementation of MSAT at the village level in a malaria-endemic area, with the screening of individuals irrespective of symptoms. To assess the impact of MSAT, studies needed to present data either collected before and after the intervention or collected in intervention and control villages.

One reviewer performed database searches using Scopus, Ovid Medline, and Web of Science using the search strategy shown in Additional File 1, Box 1. Search results were uploaded to the Covidence platform [20], where duplicate articles were identified and removed by the platform’s automated methods. One reviewer screened the titles and abstracts of all unique records, and two reviewers screened the full texts of potentially relevant studies against the inclusion criteria. Studies that were deemed ineligible were recorded alongside the reasons for exclusion. Discussions between the two reviewers were used to resolve disagreements in study inclusions with the involvement of a third reviewer when necessary. This systematic review has been registered with PROSPERO under the registration number CRD42021279109.

#### Data extraction

Using an Excel template developed by two reviewers, one reviewer extracted information from the studies included in the systematic review, including country, malaria endemicity, study design, comparison intervention, the number of MSAT rounds conducted, population studied, diagnosis method, treatment provided to positive cases, and the outcome measure and effect measure.

#### Synthesis of results

Studies were summarized according to the study design, and the comparator used to assess the impact of MSAT. A random-effects meta-analysis was performed in R using the metafor package [21], with studies grouped according to study design and comparator used. The between-study heterogeneity was examined using Cochrane’s Q test and quantified with the (*I*^*2*^) value, which measures the percentage of the total variation in results across studies due to heterogeneity. The effect measure obtained from the analysis of METF data was included in the meta-analysis as the Incidence Rate Ratio (IRR) comparing incidence in the year prior to MSAT with the incidence in the two years post-MSAT.

#### Bias assessment

Two reviewers assessed the risk of bias for the included studies using the ROBINS-I tool [22] for non-randomized studies and the ROB2 tool [23] for randomized studies. Discussions between the two reviewers resolved disagreements in the bias assessment, with the involvement of a third author when necessary.

## Results

### MSAT in the METF programme

In 2018, MSAT was delivered in 10 villages, during which 1,248 individuals were tested using uRDTs. This corresponds to 94.1% (1248/1326) of the population screened according to census information collected prior to MSAT. Of the individuals tested, 12 *P. falciparum* cases were diagnosed and treated, corresponding to an overall test positivity of 0.96% (village-level positivity ranged from 0 to 3.4%) and a median (25 th–75 th percentile) village-level *P. falciparum* test positivity of 0.67% (0.1–1.5%). Standard RDTs detected no *P. falciparum* cases detected by uRDTs (0/12). MSAT was conducted on different dates across the 10 villages in 2018. However, all malaria posts had at least 34 months of pre-MSAT and 36 months of post-MSAT incidence data.

The monthly incidences of *P. falciparum* and *P. vivax* were also lower in the years preceding MSAT when compared to the year MSAT was delivered (Table 1). This reflects the unpredictable nature of the increase in incidence, which was the trigger for MSAT delivery. The monthly incidence of *P. falciparum* decreased by 63% (95% CI: 81–27% decrease) in the year after MSAT. This was in line with an overall decrease in *P. falciparum* incidence across the METF malaria post network of malaria posts over the same period, coinciding with the end of 2018 to 2021 (see Additional File 2, Figure S1). In the year following MSAT delivery, there was also a 40% decrease (95% CI: 55–20% decrease) in *P. vivax* monthly incidence in the year after MSAT when compared with the year prior to MSAT, despite *P. vivax* malaria cases not receiving radical cure during this period (Table 1).

**Table 1:** Relative change in monthly *P. falciparum* and *P. vivax* incidence by years since MSAT

Covariate		*P. falciparum*	*P. vivax*
		IRR (95% CI)	
Years since MSAT	−3	0.52 (0.26, 1.02)	0.36 (0.26, 0.51)
	−2	0.78 (0.42, 1.43)	0.31 (0.22, 0.43)
	−1	Reference	Reference
	0	0.37 (0.19, 0.73)	0.60 (0.45, 0.80)
	1	0.01 (0.00, 0.05)	0.52 (0.38, 0.70)
	2	0.01 (0.00, 0.04)	1.37 (1.03, 1.81)
	3	0.21 (0.08, 0.54)	2.07 (1.53, 2.81)

Mixed-effects negative binomial modelling with a random intercept for malaria post and random slope (linear and quadratic) for years since MSAT. Seasonality was captured using 3 Fourier terms per year

*CI* confidence interval, *IRR* Incidence Rate Ratio, *MSAT* mass screen and treatment

**Fig. 2:**
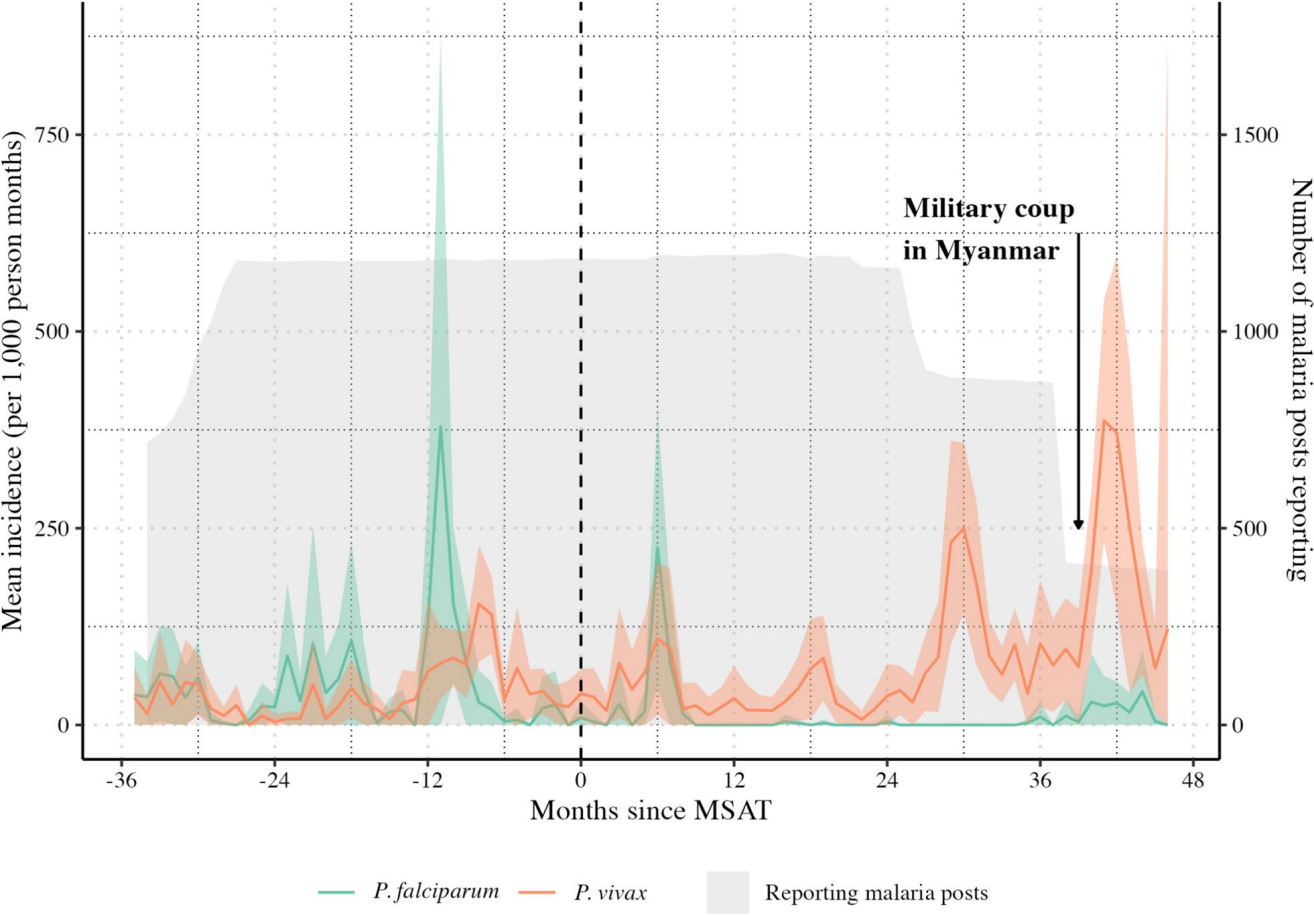
Mean monthly incidences of *P. falciparum* and *P. vivax* by months since MSAT intervention. The mean monthly incidence of *P. falciparum* (*green line*) and *P. vivax* (*orange line*) with corresponding 95% confidence intervals calculated for the METF malaria posts where mass screening and treatment (MSAT) was delivered according to the number of months since MSAT. The total number of malaria posts providing malaria services and providing weekly data is shown in *grey*. Data are centred around the date of MSAT, as indicated by the *vertical line*

An increase in *P. falciparum* incidence at the six-month time point after MSAT was representative of an increase in seven (70%) malaria posts, where this period coincided with the wet season transmission peak in 2019 (Fig. 2). The increase in *P. falciparum* and *P. vivax* incidence after the third year post-MSAT coincides with an increase in malaria transmission in seven malaria posts following the military coup in Myanmar in February 2021 (Fig. 2), which resulted in changes in population movement fluctuations in Karen State. The mean monthly *P. falciparum* incidence for each malaria post that received MSAT separately is shown in Additional File 2, Fig. S2.

### Systematic review and meta-analysis

#### Description of studies

**Fig. 3:**
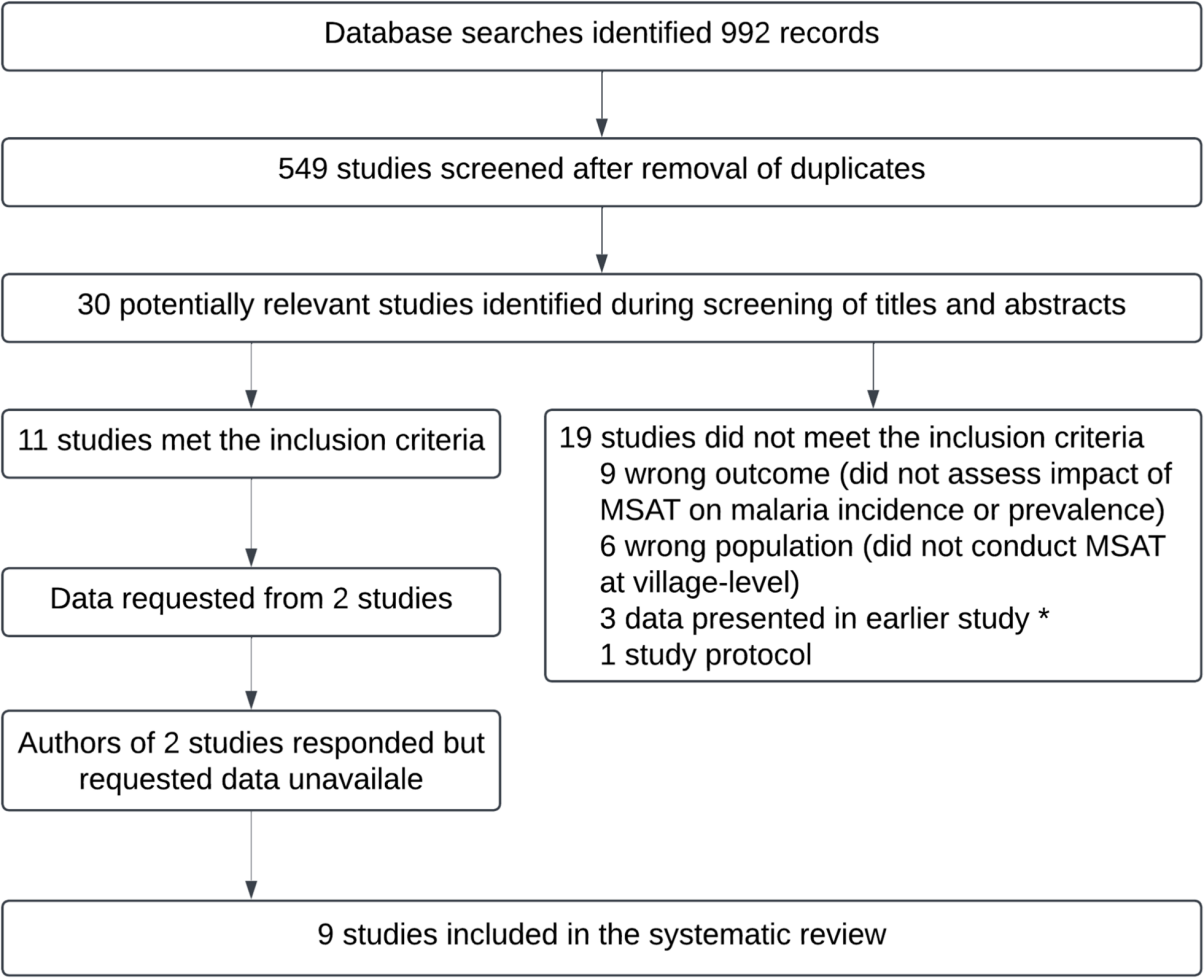
Flow diagram of village-based mass screening and treatment (MSAT) studies identified during screening. *Studies were excluded if data were already presented in an included study which was published at an earlier date

After screening the titles and abstracts of 549 articles published up until the 11 th of July 2022, the full texts of 30 potentially relevant studies were reviewed, of which nine were included in the systematic review (Fig. 3) and are summarized in Table 2. The studies included in this review were conducted across seven countries (six African countries and Indonesia) of varying malaria endemicity. The combined results of these studies are representative of more than 120 rounds of MSAT, with a median of 3 (range: 1–85) MSAT rounds conducted in each study. In the studies which reported the results of screening (all except Larsen et al*.* [24]), 240,810 individuals were tested, resulting in the diagnosis of 74,756 *P. falciparum* cases, which corresponds to an overall positivity rate of 31% and a mean (range) positivity rate per study of 9.9% (0.2–41.5%).

**Table 2:** Summary of studies included in the systematic review

Study	First author, year	Study period	Country	Malaria endemicity^a^	Study design	Study population	Comparator	Assessment of outcome measure	Number of MSAT rounds	Percent of target population screened (%)^b^	Diagnosis method	Treatment	Outcome measure	Effect measure	Positivity rate during MSAT (%)^c^
1.1	Sutcliffe, 2012 [25]	2007	Zambia	Low/moderate	Community RCT	General population	Control–single MSAT round	Cross-sectional survey^d^	1–4	Not stated	RDT	AL	Prevalence	OR	19.5
1.2	Sutcliffe, 2012 [25]	2008–2009	Zambia	Low/moderate	Community RCT	General population	Control–single MSAT round	Cross-sectional survey^d^	1–11	Not stated	RDT	AL	Prevalence	OR	2.4
2	Mlacha, 2020 [28]	2015–2018	Tanzania	Moderate/high	CnRCT	General population	Control–standard of care	Cross-sectional survey	85^e^	53	RDT	DP	Prevalence	OR	24.6
3	Larsen, 2015 [24]	2011–2013	Zambia	Low/moderate	Stepped-wedge community RCT	General population	Control–standard of care	Passive case detection	3	88	RDT/microscopy	AL	Incidence rate	IRR	8.5–23.2^f^
4.1	Desai, 2020 [26]	2013–2015	Kenya	High	CRCT	General population	Control–standard of care	Passive case detection	6	75–94	RDT/microscopy	DP	Incidence rate	IRR	41.5
4.2	Desai, 2020 [26]	2013–2015	Kenya	High	CRCT	General population	Control–standard of care	Cross-sectional survey	6	75–94	RDT/microscopy	DP^g^	Incidence rate^h^	IRR	41.5
5	Bahk, 2018 [32]	2015–2017	Uganda	High	Longitudinal cohort	General population	Pre-intervention	Cross-sectional survey^d^	6^e^	31–56	RDT	Not stated	Prevalence	PR^i^	20.5
6	Cook, 2015 [29]	2012	Zanzibar	Low	CnRCT	General population	Pre-intervention	Cross-sectional survey	1^j^	53	RDT	ASAQ	Prevalence	PR^i^	0.2
7.1	Searle, 2021 [31]	2016–2018	Zambia	Low	Longitudinal cohort	Index household	Pre-intervention	Cross-sectional survey^d^	2^k^	68–100	RDT	AL	Prevalence	PR^i^	1.2
7.2	Searle, 2021 [31]	2016–2018	Zambia	Low	Longitudinal cohort	Neighbour of index case (<140 m)	Pre-intervention	Cross-sectional survey^d^	2^k^	59–100	RDT	AL	Prevalence	PR^i^	0.6
7.3	Searle, 2021 [31]	2016–2018	Zambia	Low	Longitudinal cohort	Neighbour of index case (140–250 m)	Pre-intervention	Cross-sectional survey^d^	2^k^	54–100	RDT	AL	Prevalence	PR^i^	0.5
8.1	Sutanto, 2018 [27]	2013	Indonesia	Moderate/high	CRCT	General population	Pre-intervention	Cross-sectional survey^d^	2	86–91	Microscopy	DP	Prevalence	PR^i^	4.2
8.2	Sutanto, 2018 [27]	2013	Indonesia	Moderate/high	CRCT	General population	Pre-intervention	Cross-sectional survey^d^	3	82–92	Microscopy	DP	Prevalence	PR^i^	2.2
9	Conner, 2020 [30]	2014–2015	Senegal	Low/moderate	CnRCT	General population	Pre-intervention	Passive case detection	1	86	RDT	DP	Incidence rate	IRR	1.5

*MSAT* mass screening and treatment, *CRCT* cluster randomized controlled trial, *CnRCT* cluster non-randomized controlled trial, *RCT* randomized controlled trial, *RDT* rapid diagnostic test, *AL* artemether-lumefantrine, *DP* dihydroartemisinin piperaquine, *ASAQ* artesunate-amodiaquine, *OR* odds ratio, *IRR* incidence rate ratio, *PR* prevalence ratio

Incidence rate calculated as cases per person year

^a^Provided by authors of the study

^b^Overall population screened, or range of population screened in studies where estimates are provided by MSAT round

^c^Positivity rate of *P. falciparum* detected during MSAT interventions may include multiple rounds of MSAT

^d^Multiple cross-sectional surveys were conducted to assess MSAT. Prevalence estimates combined over the post-intervention period

^e^Not all intervention rounds conducted in all villages

^f^Village-level positivity given as a percentage. No raw numbers were provided

^g^All positive individuals treated with a full course of AL at enrolment

^h^Active screening conducted on a random subset of the population included intervention and control clusters in study 4.1. Unclear what denominator was used in the calculation of incidence

^i^Effect measure calculated from prevalence/incidence estimates. Does not include adjustment for other covariates

^j^Two MSAT rounds were conducted, but the estimated post-MSAT prevalence was collected after only the first round

^k^MSAT was conducted at an initial visit (day 0) and 30 and 90 days, resulting in prevalence estimates collected after the first two MSAT rounds

Four studies were randomized controlled trials [24–27], three were non-randomized controlled trials [28–30], and two were longitudinal cohorts [31, 32]. Of the four randomized controlled trials, three randomly selected communities or community clusters to receive MSAT or the standard of care [24, 25, 27], whereas in the study by Desai et al*.* intervention and control clusters were purposively selected based on criteria including malaria burden and access to road networks for the transport of samples in the study [26, 33]. In the three non-randomized controlled trials, high- and low-incidence clusters were included in both intervention and control arms [28, 29], or MSAT was delivered in high-incidence clusters only [30].

Limited information was provided on the age and sex of the participants included in MSAT rounds; however, in the five studies that did report the sex distribution of participants, approximately 50% were male [25–28, 31]. In the study by Sutcliffe et al*.*, the majority of individuals screened were less than 15 years of age [25]. In the studies by Mlacha et al*.* and Desai et al*.* [26, 28] (only study 4.2 in Table 2), approximately 50 and 40% were less than 15 years of age, respectively, while in the study by Searle et al*.* [31], the maximum age of surveyed individuals was 33 years of age. No details were provided on the number of individuals within each age bracket in the study by Searle et al*.* Since the included studies did not provide census information, it was not possible to determine whether the age and sex distributions of the study population were representative of the actual population.

In seven of the nine studies, multiple rounds of MSAT were conducted either during consecutive months [24, 26, 27, 29], within a defined time period [24, 25, 32], or in response to the identification of *P. falciparum* cases during passive case detection [28, 31]. In the majority of studies (67%, 6/9), MSAT was conducted during household visits [24–26, 29–31]. However, this did not result in higher coverage rates compared with studies that conducted MSAT at a location within the village where everyone was screened [27, 28, 32] (Table 2).

The impact of MSAT was assessed either by comparing post-MSAT *P. falciparum* incidence in intervention and control villages [24, 26, 30], by comparing post-MSAT *P. falciparum* prevalence estimates collected during cross-sectional surveys in intervention and control villages [25, 28], or by comparing *P. falciparum* prevalence before and after MSAT using cross-sectional surveys [27, 29, 31, 32]. In four of the nine studies, multiple measurements of MSAT impact were presented, resulting in a total of 14 effect measurements recorded across the included studies (Table 2). In the studies by Sutanto et al*.* and Sutcliffe et al*.*, a different number of MSAT rounds were conducted in two population cohorts [25, 27]; in the study by Desai et al., the impact of MSAT was assessed either through passive case detection or active case detection [26]; and in the study by Searle et al*.*, the impact of MSAT on *P. falciparum* prevalence at varying distances from index households was investigated [31].

Across the nine studies, the impact of MSAT was measured after differing time intervals. In the study by Sutcliffe et al*.*, *P. falciparum* prevalence was measured only once (before MSAT) in households in the cross-sectional cohort which acted as the control and was measured multiple times in the longitudinal cohort at the time of MSAT delivery over a one (study 1.1) or two-year period (study 1.2) [25]. In the study by Mlacha et al*.*, *P. falciparum* prevalence was measured in a random subset of the population using cross-sectional surveys before and two and a half years after MSAT. In studies by Desai et al*.* and Larsen et al*.*, passive case detection was conducted over a one-to-two-year period following intervention delivery in intervention and control villages through the network of local health facilities. In studies which compared *P. falciparum* prevalence before and after MSAT delivery, the follow-up period was between 30 and 90 days for all studies [27, 29, 31] except for the study by Bahk et al., which assessed the impact of MSAT in a follow-up survey two years after MSAT [32].

In several studies, multiple outcome measures were collected; however, relevant data were only provided for some. In the study by Sutanto et al*.*, follow-up data was not presented for the control clusters where village-level screening was not conducted [27]. Accordingly, the prevalence estimate from the pre-MSAT period was used as the comparator for the Sutanto et al*.* study in this review. In Cook et al*.*, passive case detection and cross-sectional surveys were used to assess the impact of MSAT; however, *P. falciparum* incidence measurements were not provided in the results of this study (incidence only shown graphically) [29]. For the study by Larsen et al*.*, the impact of MSAT was assessed using cross-sectional surveys and passive case detection, but results were presented for passive case detection only (authors only provide the percent change in prevalence before and after MSAT from survey data) [24].

In eight of the nine studies, insecticide-treated nets were distributed either before programme commencement [24–26], as part of the study [27, 28], or by a different organization during the study [29–31]. The dominant vector species was mentioned in seven of the nine studies and included *Anopheles gambiae* [26–28], *Anopheles funestus* [25, 26, 30, 32], *Anopheles arabiensis* [24, 25, 30, 31], and *Anopheles barbirostris* [27].

#### MSAT study results and meta-analysis

**Fig. 4:**
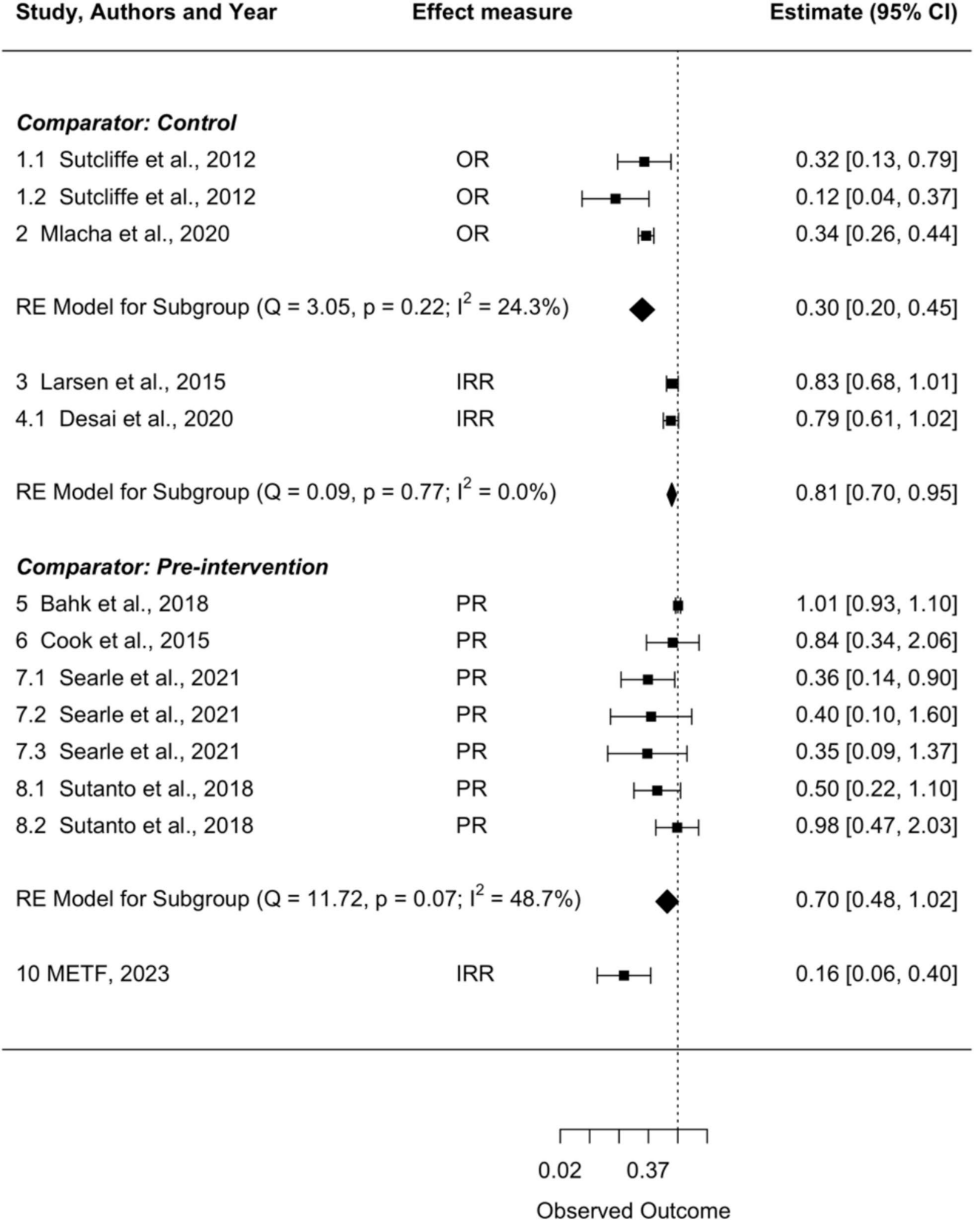
Impact of MSAT interventions on *P. falciparum* incidence and prevalence. Random-effects (RE) meta-analysis model for studies grouped according to the comparator and effect measure used to assess the impact of mass screening and treatment (MSAT) on *P. falciparum* incidence, using incidence rate ratio (IRR), or *P. falciparum* prevalence, using the odds ratio (OR) or prevalence ratio (PR). Multiple estimates were included for studies which delivered or measured the impact of MSAT using multiple methods

To compare the impact of MSAT interventions across the studies included in this review, a meta-analysis was performed on studies grouped according to the comparator and the effect measure used in the assessment of MSAT. To allow for the comparison of METF results with results from the other studies using the pre-intervention period as the comparator, METF results were included in the meta-analysis as the incidence rate ratio comparing the incidence in the year prior to MSAT with the incidence in the two years post-MSAT. The meta-analysis included all studies except for the study by Conner et al*.*, in which MSAT was conducted alongside other interventions so the effect measure (IRR = 0.62, 95% CI: 0.45, 0.84) cannot be used to assess the impact of MSAT alone [30], and the study by Desai et al*.* (study 4.2) in which active screening was performed on a random subset of the same population analysed during passive case detection in study 4.1 [26]. All other studies with two or more intervention arms assessed the impact of MSAT in separate sub-groups of the target population [25, 27, 31]. Slight differences between the confidence intervals reported in Sutcliffe et al*.* and those reported in Fig. 4 are a result of rounding errors.

In the two randomized controlled trials which assessed the impact of MSAT using cross-sectional surveys, the pooled Odds Ratio (OR) shows a reduction in the odds of *P. falciparum* positivity in villages that received MSAT when compared with control villages (OR = 0.30, 95% CI: 0.20, 0.45, *I*^*2*^ = 24.3%) [25, 28] (Fig. 4). In the study by Sutcliffe et al*.*, there was a greater reduction in the odds of *P. falciparum* positivity following MSAT in the 2008/2009 cohort (OR = 0.12, 95% CI: 0.04, 0.37) when compared with the 2007 cohort (OR = 0.32, 95% CI: 0.13, 0.79) where the baseline prevalence of *P. falciparum*, measured at the first study visit prior to MSAT delivery, was higher for the 2007 cohort (Table 2).

In the two cluster randomized controlled trials which assessed the impact of MSAT through passive case detection, the pooled IRR shows a reduction in *P. falciparum* incidence in the villages that received MSAT when compared with control villages one-to-two-year post-MSAT (IRR = 0.81, 95% CI: 0.70, 0.95, *I*^2^ = 0.0%) (Fig. 4) [24, 26]. In study 4.2 by Desai et al*.* (excluded from the meta-analysis), there was a decline in *P. falciparum* incidence following MSAT during cross-sectional surveys in a random subset of the population included in study 4.1 (IRR = 0.95, 95% CI: 0.87, 1.04) [26].

The pooled Prevalence Ratio (PR) from studies which compared the post-MSAT *P. falciparum* prevalence to the pre-MSAT prevalence was 0.54 (95% CI: 0.34, 0.87, *I*^*2*^ = 72.7%). In studies by Bahk et al., Searle et al., and Sutanto et al., the effect measure was derived from the prevalence estimates provided in each of the studies for this review, so it does not account for other covariates including season and demographic factors, which may be important to consider in these studies [27, 31, 32].

In the METF analysis and studies by Mlacha et al*.* and Searle et al*.*, MSAT was deployed in villages or households in response to the diagnosis of *P. falciparum* cases at health facilities [28, 31]. These interventions resulted in a decrease in *P. falciparum* incidence in the METF area (IRR = 0.16, 95% CI: 0.06, 0.40), a reduction in *P. falciparum* prevalence in the study by Mlacha et al*.* (OR = 0.34, 95% CI: 0.26, 0.44) [28], and a decrease in *P. falciparum* prevalence in the study by Searle et al*.* (PR = 0.36, 95% CI: 0.14, 0.90) at the households of index cases, but not at the households within a 250 m radius of the index households (Table 2) [31].

Comparisons between studies with different *P. falciparum* positivity rates and proportions of the population screened during each round of MSAT did not reveal any clear relationships between these factors and the resulting impact of MSAT (Table 2 and Fig. 4).

#### Risk of bias assessment

All studies included in the meta-analysis (Fig. 4) underwent a risk of bias assessment, with studies grouped according to whether the delivery of MSAT was randomized (see Additional File 3, Table S2 and S3). In the non-randomized trials, there were concerns about the selection of participants in two studies, Bahk et al*.* and the METF study, because MSAT was delivered at several time points, resulting in variable amounts of follow-up time, which were not accounted for in the analyses [32]. The risk of confounding in non-randomized trials was assessed as low based on the fact that confounding was explicitly addressed in these studies [22]. However, residual confounding, particularly due to unmeasured time-varying confounding and the small number of clusters included in some studies, remains a possibility. In the studies by Mlacha et al*.* and Bahk et al*.*, the impact of MSAT was assessed in a random subset of between 31 and 56% of the population (different coverage between MSAT rounds), meaning the outcome measure was missing for much of the target population [28, 32]. 

In two of the four randomized controlled trials [24, 25], individuals were recruited following the randomization of clusters to intervention and control arms. In the study by Sutcliffe et al., this is unlikely to have had an impact on the recruitment of individuals into the study because MSAT was delivered at least once in both study arms [25], whereas in the study by Larsen et al., it is less clear whether this would have affected recruitment [24], so there are some concerns around the recruitment process in this study. Additionally, in the study by Sutcliffe et al., there were differences in insecticide-treated net ownership and usage and treatment-seeking behaviour between the intervention and control arms [25], and in the study by Larsen et al., there were differences in insecticide-treated net ownership and indoor residual spraying in the previous 12 months between the intervention and control arms [24]. These differences could indicate issues in the randomization process and may have influenced the impact of MSAT in these studies. More information on the distribution of households and the randomization process would help alleviate doubts about the randomization process in these studies.

## Discussion

This study presents a retrospective analysis of 10 single-round MSAT interventions conducted at the village level by the METF programme in Eastern Myanmar alongside a systematic review and meta-analysis of nine studies of village-level MSAT interventions. In the METF programme, an overall reduction in *P. falciparum* incidence was seen in the 10 villages that received MSAT. However, the continued availability and uptake of early diagnosis and treatment services provided by the METF malaria posts is likely the major contributing factor in the decline of *P. falciparum* incidence at the villages which received MSAT, as region-wide declines in *P. falciparum* incidence were observed over the period in which MSAT was administered and assessed. A limitation of the METF analysis is that no control group was selected during MSAT to act as the comparator.

While all studies included in the meta-analysis (METF study results and eight studies identified during the systematic review) reported a reduction in the incidence or prevalence of *P. falciparum* following MSAT, the magnitude of the impact differed greatly and was likely influenced by a variety of factors including the coverage of MSAT in the target population, the baseline *P. falciparum* endemicity, and the methods used in MSAT delivery and assessment. This highlights the variable impact of MSAT in different settings.

The proportion of *P. falciparum* cases diagnosed and treated during MSAT rounds is a key factor in the impact of this intervention—the greater the proportion of *P. falciparum* cases detected and treated, the greater the reduction in the reservoir of infections remaining in the community. While MSAT and active screening interventions, such as the “1–3–7” approach, have been suggested for low transmission areas, low positivity rates and the limited diagnostic sensitivity of RDTs and uRDTs inherently limit the impact of these strategies [34, 35]. This was also identified as a limitation in several studies included in this review [24, 26, 32]. It is, therefore, essential that a large proportion of the target population is screened to detect as many *P. falciparum* cases as possible during MSAT. In the METF programme, while the overall detection of malaria infections was low, using uRDTs during MSAT increased the number of *P. falciparum* cases detected compared to standard RDTs. However, the ability of uRDTs and RDTs to detect the majority of cases depends on the proportion of low-density parasite infections in the community [35].

In several studies, only 50% of the target population was screened in one or more MSAT rounds [28, 29, 31, 32]. The reasons for low MSAT coverage provided by these studies were absenteeism at the time of screening [29, 31], insufficient community engagement before repeated screening rounds [29], or screening limited to a random subset of the population [28, 32]. While screening was done during household visits in the majority of studies [24–26, 29–31], this did not impact MSAT coverage when compared with studies in which individuals attended screening at a location within the village [27, 28, 32].

In the studies by Mlacha et al*.* and Searle et al*.*, MSAT was delivered in response to identifying *P. falciparum* cases at health facilities in the target area [28, 31]. In these studies, there was a reduction in *P. falciparum* prevalence in the households of index cases following MSAT, but not in households within a 250 m radius of index households [31] where population coverage during screening was lower. Based on the studies included in this review, there is no clear relationship between the coverage of MSAT or positivity rate and the reduction in *P. falciparum* prevalence or incidence. While both factors play a role in MSAT impact, their importance likely depends on context-specific factors, including access to early diagnosis and treatment, the prevalence of asymptomatic infections, adherence to prescribed antimalarials, and demographic factors.

Demographic information, including the gender and age of individuals in the intervention, can provide insight into possible reasons for low intervention coverage. Of the studies that did provide demographic information, there was an almost equal distribution of females and males. However, the age distribution suggests limited coverage in older individuals [25, 27, 29, 30], which may indicate limitations in the delivery of MSAT in these studies.

In four studies, including the METF programme [27, 31, 32], *P. falciparum* incidence or prevalence measured after MSAT was compared to measurements collected prior to MSAT. In these studies, it is difficult to distinguish between the impact of MSAT and the decline in incidence over time, which would have occurred in the absence of MSAT. For example, in the METF programme, there was an overall decline in *P. falciparum* incidence in the METF programme area over the period coinciding with the post-MSAT period [12], which is likely the driver of the decline in MSAT villages as well.

One limitation of this review is that it was not possible to discern what impact individual factors had on the success of MSAT due to the wide range of methods used in MSAT delivery and differences in the study areas where MSAT was delivered. Another limitation is that due to differences in the comparator and outcome measures used, it was not possible to compare and pool the effect of MSAT across all studies. When used alone, prevalence and incidence measures of intervention impact both have limitations. Prevalence estimates provide only a snapshot of the malaria burden and, depending on malaria seasonality may overestimate or underestimate the impact of interventions. On the other hand, the treatment-seeking behaviour of the study population influences incidence measurements collected during passive case detection. To provide more reliable estimates of the impact of population-level malaria interventions, a combination of prevalence measured using repeated cross-sectional surveys and incidence measured through passive case detection should be used to compare intervention and control villages matched on a range of factors, including baseline malaria incidence or prevalence.

## Conclusion

This retrospective analysis of MSAT interventions conducted by the METF programme, presented alongside a systematic review and meta-analysis of other studies evaluating the impact of village-based MSAT interventions, reveals the complexities behind the success of targeted interventions for malaria elimination. In the METF programme, the overall decline in *P. falciparum* incidence across the malaria post network was the likely driver of the decline in incidence following MSAT administration in the 10 villages that received MSAT. Across a variety of endemicities, the nine MSAT studies identified in the systemic review demonstrated a general reduction in *P. falciparum* incidence and prevalence following MSAT. The magnitude of this impact differed between studies, likely influenced by a wide range of factors from baseline endemicity, population demographics, and the timing and uptake of the intervention.

## Supplementary Information

Additional file 1: Box 1. Search strategy for systematic review.

Additional file 2: Fig. S1. Mean monthly incidence ofP. falciparum andP. vivax across all METF malaria posts. Fig. S2. Mean monthly incidence for P. falciparum by months since MSAT intervention at METF malaria posts.

Additional file 3: Table S2. Risk of bias assessment performed using ROBINS-I for non-randomized trials. Table S3. Risk of bias assessment performed using ROB-2 for randomized trials.

## Data Availability

The data analysed for this study are available upon request to the Mahidol-Oxford Tropical Medicine Research Unit data access committee: https://www.tropmedres.ac/units/moru-bangkok/bioethics-engagement/data-sharing.
