# Supplementary material for: The impact of mass screening and treatment interventions on malaria incidence and prevalence: a retrospective analysis of a malaria elimination programme in eastern Myanmar, and systematic review and meta-analysis
Source: Malar J. 2025 May 8;24:148. doi: 10.1186/s12936-025-05392-9 (PMC12063463; doi:10.1186/s12936-025-05392-9)
Supplement: Supplementary file 1 — Additional file 1: Box 1. Search strategy for systematic review. [file 12936_2025_5392_MOESM1_ESM.pdf]

Additional File 1

Box 1. Search strategy

(malaria OR falciparum) AND (MSAT OR MTAT OR FSAT OR FTAT OR ACD OR RACD OR RCD OR "mass screen*" OR "mass test*" OR "foci screen*" OR "foci test*" OR "focal screen*" OR "focal test*" OR "active screen*" OR "active test*" OR "proactive screen*" OR "proactive test*" OR "reactive screen*" OR "reactive test*" OR "active case detection" OR "proactive case detection" OR "reactive case detection" OR "foci case detection" OR "focal case detection" OR "screen and treat" OR "screening and treatment" OR "test and treat" OR "testing and treatment") AND (RDT OR HSRDT OR HS-RDT OR URDT OR "rapid diagnostic test*") AND (case* OR incidence OR rate* OR prevalence OR effect* OR impact OR "rate ratio" OR "odds ratio" OR "risk ratio" OR "relative risk" OR "risk reduction" OR efficacy)
