# Supplementary material for: The impact of mass screening and treatment interventions on malaria incidence and prevalence: a retrospective analysis of a malaria elimination programme in eastern Myanmar, and systematic review and meta-analysis
Source: Malar J. 2025 May 8;24:148. doi: 10.1186/s12936-025-05392-9 (PMC12063463; doi:10.1186/s12936-025-05392-9)
Supplement: Supplementary file 2 — Additional file 2: Fig. S1. Mean monthly incidence ofP. falciparum andP. vivax across all METF malaria posts. Fig. S2. Mean monthly incidence for P. falciparum by months since MSAT intervention at METF malaria posts. [file 12936_2025_5392_MOESM2_ESM.pdf]

**Additional File 2**

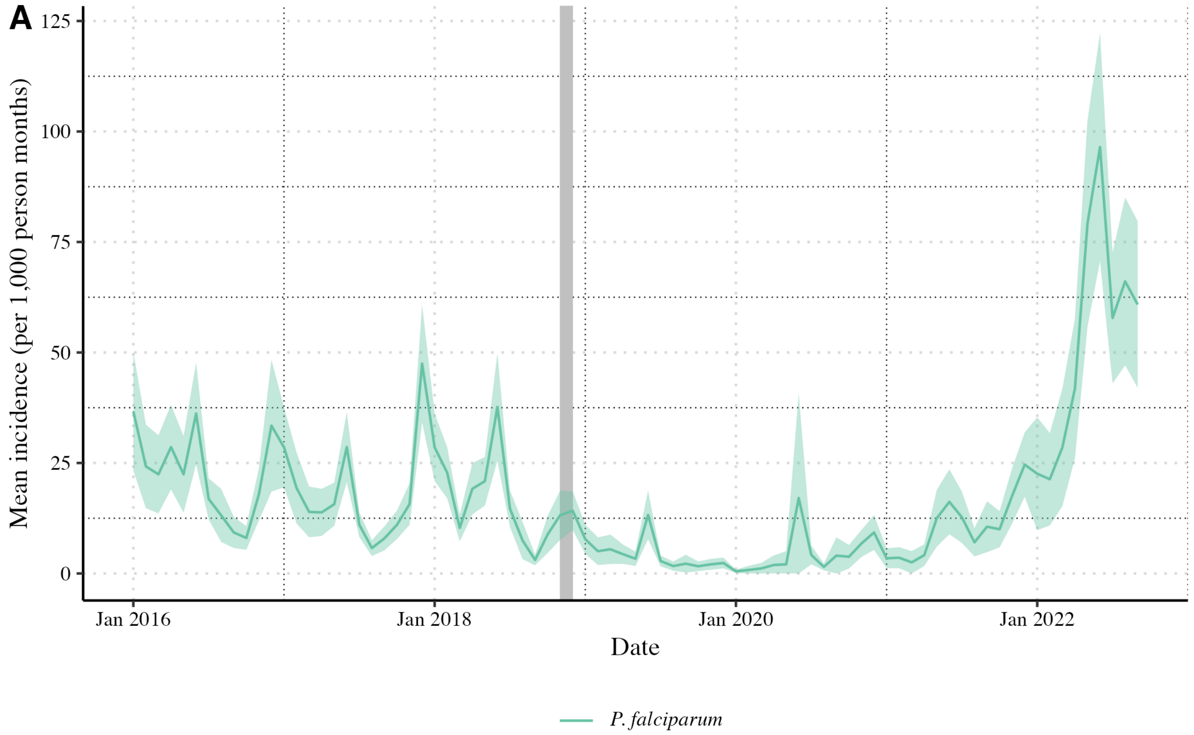


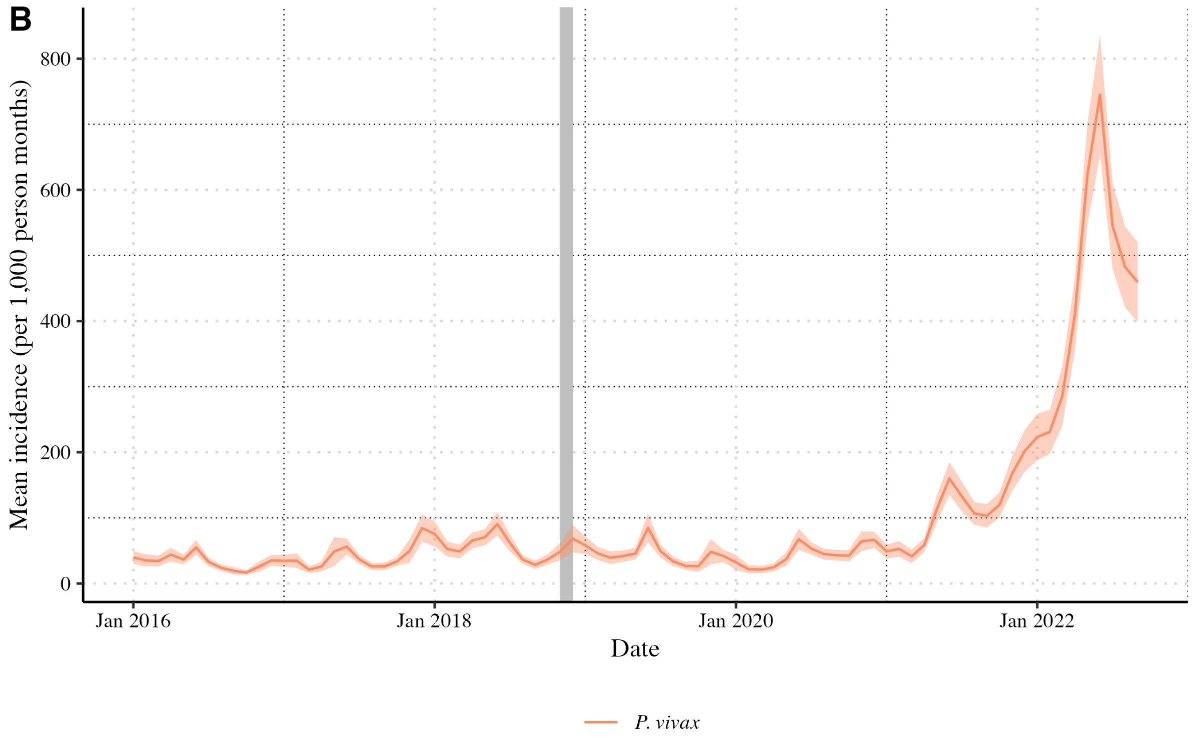


**Figure S1. Mean monthly incidence of (A) *P. falciparum* and (B) *P. vivax* across all METF malaria posts.** The mean monthly *P. falciparum* (green) and *P. vivax* (orange) incidence was calculated for all malaria posts that reported weekly surveillance data between 2016 and 2022. The grey bar shows the period during which MSAT was delivered at the ten villages that received this intervention (between November and December 2018). The scale of the y-axis differs between panels.

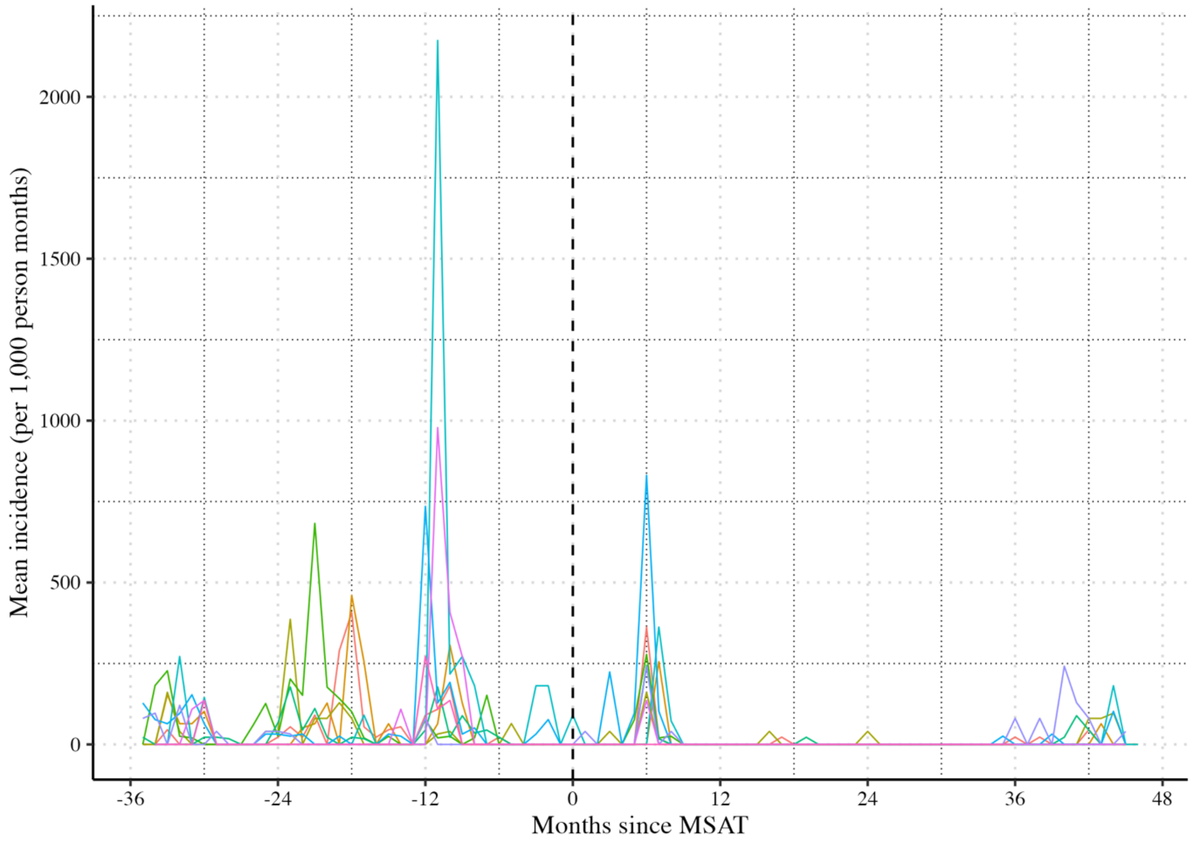


**Figure S2. Mean monthly incidence for *P. falciparum* by months since MSAT intervention at METF malaria posts.** The mean monthly *P. falciparum* incidence calculated for the METF malaria posts that received mass screening and treatment (MSAT). Incidence coloured according to malaria post. Data are centred around the date of MSAT, indicated by the vertical dashed line.
