# Supplementary material for: The impact of mass screening and treatment interventions on malaria incidence and prevalence: a retrospective analysis of a malaria elimination programme in eastern Myanmar, and systematic review and meta-analysis
Source: Malar J. 2025 May 8;24:148. doi: 10.1186/s12936-025-05392-9 (PMC12063463; doi:10.1186/s12936-025-05392-9)
Supplement: Supplementary file 3 — Additional file 3: Table S2. Risk of bias assessment performed using ROBINS-I for non-randomized trials. Table S3. Risk of bias assessment performed using ROB-2 for randomized trials. [file 12936_2025_5392_MOESM3_ESM.pdf]

Additional File 3

Table S2. Risk of bias assessment performed using ROBINS-I for non-randomized trials.

Study	First author, year	Risk of bias resulting from/due to							Overall
		Confounding[1]	Selection of participants	Classification of interventions	Deviations from intended intervention	Missing data	Outcome measure	Selection of reported result	
2	Mlacha, 2020 [28]	Low risk	Low risk	Low risk	Low risk	Some concerns	Low risk	Low risk	Some concerns
5	Bahk, 2018 [32]	Low risk	Some concerns	Low risk	Low risk	Some concerns	Low risk	Low risk	Some concerns
6	Cook, 2015 [29]	Low risk	Low risk	Low risk	Low risk	Some concerns	Low risk	Low risk	Some concerns
7	Searle, 2021 [31]	Low risk	Low risk	Low risk	Low risk	Low risk	Low risk	Low risk	Low risk
10	METF, 2023	Low risk	Some concerns	Low risk	Low risk	Low risk	Low risk	Low risk	Some concerns

[1] Low risk of bias refers to adequately addressing confounding using measured potential confounders. Of note, there is still the possibility in these non-randomized trials of bias due to unmeasured time-varying confounders (22).

Table S3. Risk of bias assessment performed using ROB-2 for randomized trials.

Study	First author, year	Risk of bias resulting from/due to					Overall
		Randomization	Deviations from intended intervention	Missing data	Outcome measure	Selection of reported result	
1	Sutcliffe, 2012 [25]	Some concerns	Low risk	Low risk	Low risk	Low Risk	Some concerns
3	Larsen, 2015 [24]	Some concerns	Low risk	Low risk	Low risk	Low risk	Some concerns
4	Desai, 2020 [26]	Low risk	Low risk	Low risk	Low risk	Low risk	Low risk
8	Sutanto, 2018 [27]	Low risk	Low risk	Low risk	Low risk	Low risk	Low risk
